# Metastasis of Colon Adenocarcinoma to Maxillary Gingiva and Palate

**DOI:** 10.22038/ijorl.2020.42693.2394

**Published:** 2020-09

**Authors:** Zohreh Dalirsani, Nooshin Mohtasham, Negin Samiee

**Affiliations:** 1 *Oral and Maxillofacial Diseases Research Center, Dental Faculty, Mashhad University of Medical Sciences, Mashhad, Iran.*; 2 *Department of Oral and Maxillofacial Pathology, Dental Faculty, Mashhad University of Medical Sciences, Mashhad, Iran.*; 3 *Department of Oral and Maxillofacial Medicine, Dental Faculty, Mashhad University of Medical Sciences, Mashhad, Iran.*

**Keywords:** Adenocarcinoma, Colon, Metastasis, Oral diagnosis, Gingiva

## Abstract

**Introduction:**

Colon adenocarcinoma is one of the foremost causes of cancer mortality. Oral metastasis of colon adenocarcinoma is, however, rare and indicates an end-stage disease process.

**Case Report:**

We report a case of a 69-year-old female with a gingival mass diagnosed with colon adenocarcinoma and liver metastasis after one year. A swelling was found in the maxillary right buccal and lingual gingiva during physical examination. Tissue biopsy revealed an intestinal metastatic adenocarcinoma. The patient underwent chemotherapy for treatment. We also reviewed all reported cases of gingival metastasis due to colon adenocarcinoma.

**Conclusion:**

Careful examination of the oral cavity in patients with the adenocarcinomas of various organs is beneficial in the early diagnosis of metastasis since the most frequent metastatic lesion of the oral cavity is adenocarcinoma.

## Introduction

Oral cavity metastasis is an uncommon malignancy with poor prognosis (1). It contributes to 1% of malignancies in the oral cavity (2) and, in most cases, implies a predominant disorder. Oral cavity metastasis has been reported as the first indication for occult cancerous tumors in distant metastatic sites in approximately 25% of cases. Jawbones are affected by metastatic lesions twice as high as the oral mucosa. The average survival rate in oral cavity metastasis is 7 months (1). The metastasis of the oral soft tissue comprises 0.1% of oral cancerous lesions, which makes it even rarer when compared to the metastasis of the oral cavity (3). 

Although oral cavity metastases from all sorts of malignant tumors are basically possible, those arising from the lungs in men and the breasts in women are the frequent ones. On the other hand, those originating from the colon are very uncommon (2). This, however, requires the implementation of more clinical studies. It also appears that inflammation could absorb metastatic cells since the gingiva is a predominant metastatic site in the oral soft tissues (1).

There are some obstacles to the diagnosis and treatment of oral metastases. They mostly arise from the lack of clinical observations (4). It is possible that early lesions, mostly gingiva, are mistaken for reactive or hyperplastic ones (1). Furthermore, soft tissue metastases, either symptomatic or asymptomatic, might resemble other benign lesions in the oral cavity, such as pyogenic granuloma, giant cell granuloma, and fibromas (5). 

As rapid progression and aggressive growth are known to be the main characteristics of cancerous tumors, histological verification, accompanied by other diagnostic approaches are essential for the establishment of a definitive diagnosis (6). Herein, we report a patient with colon adenocarcinoma metastasized to the maxillary gingiva and hard palate.

## Case Report

A 69-year-old female with a gingival mass was referred to the Department of Oral and Maxillofacial Medicine, Faculty of Dentistry, Mashhad University of Medical Sciences, Mashhad, Iran, in November 2017. The patient had noticed a lesion in the gingiva of the right upper jaw one month earlier. The lesion was characterized by rapid growth and bleeding when brushing the teeth. Although she had undergone a radical surgery for adenocarcinoma one year earlier (i.e., November 2016), liver metastasis had occurred postoperatively. A liver biopsy was performed for definitive diagnosis, and sample analysis showed a KRAS mutation. In humans, *KRAS* is recognized as the most common mutated oncogene acting as a molecular on/off switch for cell proliferation. 

The patient underwent chemotherapy with the FOLFOX regimen (oxaliplatin-5-fluorouracil-leucovorin) for liver metastasis due to colon cancer. A gingival mass was found during the inspection on the right side of the upper jaw in which the buccal and palatal gingiva of the canine was involved up to the second premolar. The mass had rubbery consistency with a telangiectatic granular surface, tender on pressure and prone to bleeding. No osteolytic lesion was observed on radiography (^Fig’s 1^,2), and no lymphadenopathy was detected with palpation in the head and neck area.

**Fig1:**
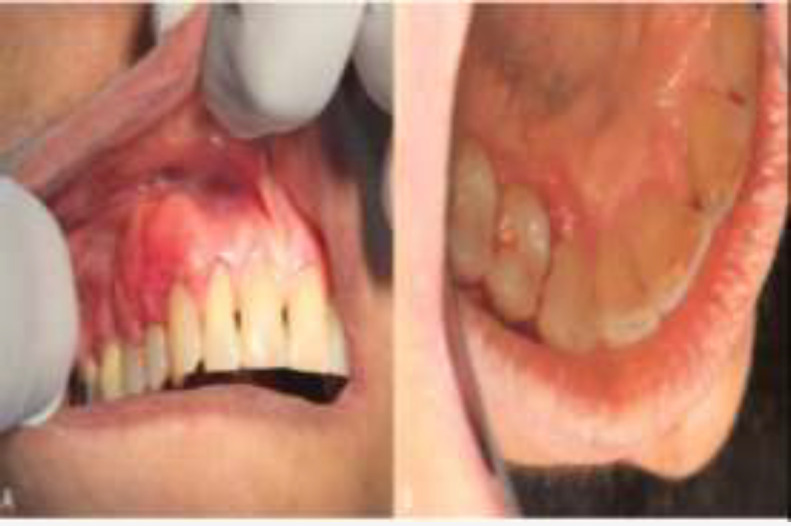
Clinical appearance of the lesion; A: Granular exophytic lesion on maxillary gingiva extended from right canine to second premolar,B: extention of the lesion to palatal interdental papillas.

**Fig 2:**
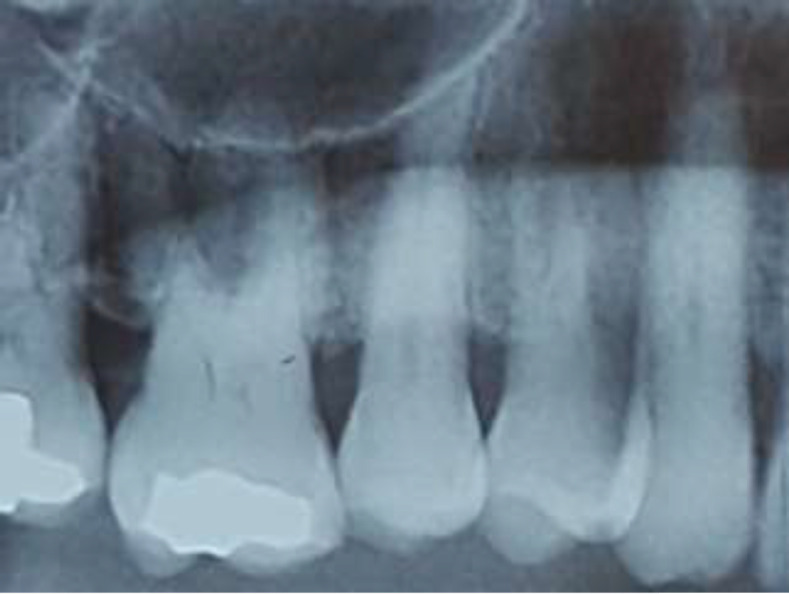
No osteolytic lesion on radiography

**Fig 3:**
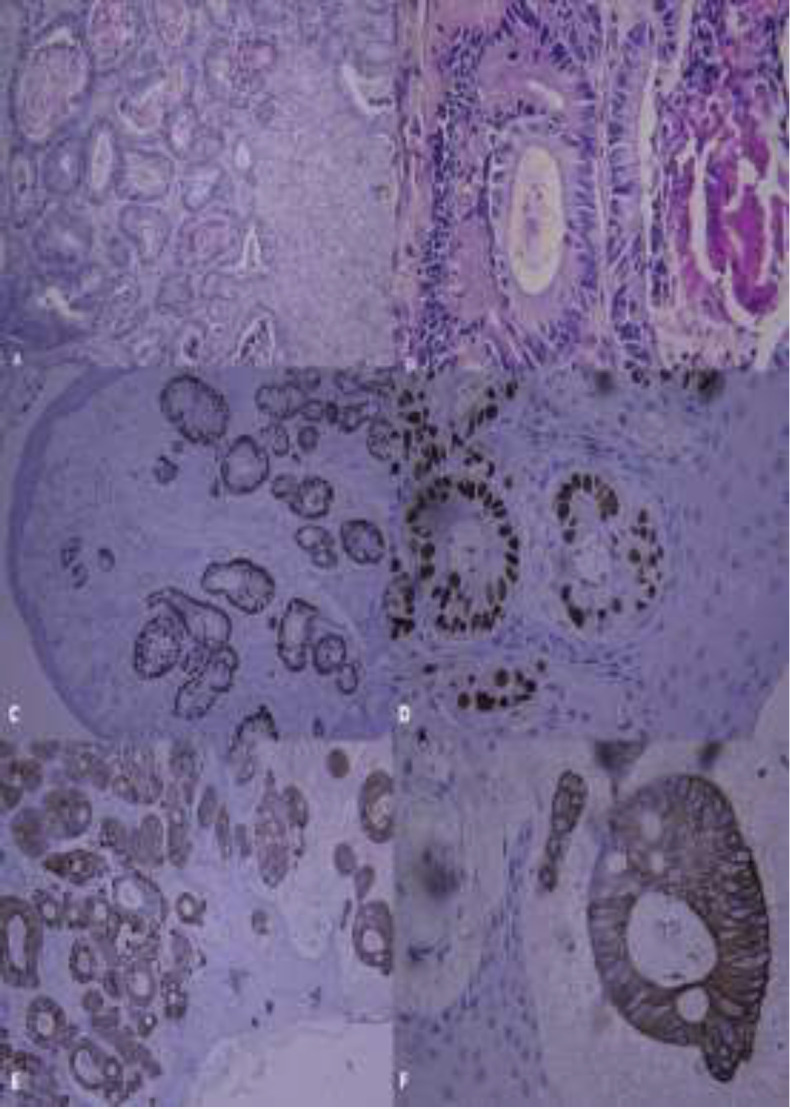
(A-B) Photomicrograph of the gingival metastatic lesion; H&E staining,100x and 400x magnification.(C-D) CK20 marker expression; IHC, 40x and 10xmagnification. (E-F) CDX2 marker expression; IHC, 40x and 10x magnification

A biopsy was performed in which the hemotoxylin and eosin staining showed the neoplastic malignant proliferation of secretory epithelial cells, which consisted of atypical glands and ducts lined by neoplastic columnar cells. Clear cells with foamy cytoplasm and goblet cells, as well as necrosis, were observed in intraductal regions. There were numerous pleomorphic, hyperchromatic cells in the fibrocellular chronic inflamed connective tissue. Immunohistochemical analysis showed immunoreactivity to markers CK20 and CDX2, as well as morphological findings. Therefore, the diagnosis of metastatic colon adenocarcinoma was confirmed (Fig.3). 

The patient was planned to continue 12 cycles of chemotherapy with the FOLFOX regimen. However, she did not respond to the treatment and passed away 6 months after being diagnosed with gingival metastasis. 

## Discussion

In this study, the relevant literature was reviewed through a bibliographic search in several databases, including the PubMed, Web of Science, and SCOPUS, without any time restriction. Only articles published in English were included in the study. 

A total of 18 cases (including the present one) with the metastatic lesions of colon adenocarcinoma on gingival mucosa were reviewed. Out of the 18 cases, 12 and 4 cases were on the mandibular gingiva and maxillary gingiva, respectively. However, no location was determined for the remaining two cases. Most of the cases with gingival metastatic lesion were previously diagnosed with colon malignancy (77%). Based on the observations, there was only one reported case of a gingival metastatic lesion as the first sign for the diagnosis of primary colon adenocarcinoma. Cases of rectal adenocarcinoma were not considered.

 Oral cavity metastases commonly appear with a prevailing disease. Their evolvement can, therefore, be considered a consequent development from other sites, particularly the lungs. However, oral metastasis has been the ﬁrst indication of occult cancerous tumors in distant metastatic sites in approximately 25% of cases. Jawbones are affected by metastatic lesions twice as high as the oral mucosa (1). Moreover, metastatic lesions influence the attached gingiva (60%) and tongue (18%) more than any other sites in the oral soft tissues. 

It could also be the case that chronic gingival inflammation provides an environment appropriate for the colonization and proliferation of metastatic cells (1). Observation of good oral hygiene can, therefore, help prevent gingival metastases. Furthermore, there are gender-based differences regarding the primary sites of the metastatic lesions. In this regard, the lungs, prostate, kidneys, and liver are the most common primary organs in men, while the primary sites in women are the genital organs, breasts, kidneys, colon, and rectum. 

Oral cavity metastases occur in patients within the age range of 50-80 years, with a mean age of 54 years (1). In our review, 55% of the patients were male. The reviewed patients had an age range of 35-85 years with the total mean age of 64.7 years, where the mean age of oral metastasis in females (67.25 years) was higher than that in males (62.8 years; Table.1). The determining role of activating the mutations of the RAS oncogenes (e.g., KRAS, HRAS,NRAS)

in the formation of cancer has become apparent as they appear in numerous cancerous tumors. Cells proliferate normally under the control of the KRAS gene. Upon the mutation of this gene, unrestricted cell proliferation due to the disruption of negative signaling evolves into malignant tumors (7).

Moreover, KRAS mutation commonly leads to malignant progression and poor response to treatment in colon cancer as observed in our case. Nevertheless, the metastasis of colon cancer to the oral soft tissue is an uncommon event. Colon cancer metastasizes to the mandible and gingiva more than to any other sites in the oral cavity; accordingly, this contributes to 25% of all oral metastases. Upon the diagnosis of colon cancer, especially in the elderly, it is recommended to pay attention to oral symptoms, such as gingival bleeding, ulcerations, and abscesses (4). From a histological perspective, adenocarcinoma accounts for almost 70% of oral metastatic lesions. Although salivary gland tumors are very uncommon in the gingiva, the primary intraoral tumors, in particular those arising from the salivary glands and squamous cell carcinoma, are the major differential diagnoses in metastatic adenocarcinoma (8). 

Therefore, the establishment of a definitive diagnosis essentially requires histological and immunohistochemical analyses. Regarding this, some baselines must be considered during the diagnosis of metastatic tumors. These baselines include the histological examination of the primary tumor, examination of the subtype identicality of the primary and metastatic tumors, and ruling out of the cases directly developed from the primary tumor (3,9,10).

**Table1:** Case studies on gingival metastasis from colon adenocarcinoma

No								
**1**	Delfino/1982	m/65	Man.	Yes	Colon to oral/ 9 months later	Surgery/CT	Died/ND	(11)
**2**	Alvarez/2006	m/62	Man.	Yes	Colon to oral/ 6 months later	None	Died/ 9 months later	(12)
**3**	Seoane/2009	f/70	Max.	ND	Colon to oral/ND	ND	Died/ND	(9)
**4**	Shen/2009	f/75	ND	ND	ND	ND	ND	(13)
**5**	Rosbottom/2009	m/73	Man.	Yes	Colon to oral/ 3 years later	Palliative RT	Died/ND	(14)
**6**	Favia/2010	f/66	Man.	Yes	Colon to oral/15 years later	Surgery	ND	(15)
**7**	Favia/2010	f/35	Man.	Yes	Colon to oral/12 months later	Surgery/CT/ Palliative RT	ND	(15)
**8**	Soares/2011	m/42	Man.	No	Oral to colon/SD	CT	Alive/ND	(8)
**9**	Jham/2011	m/53	ND	ND	ND	Surgery/CT	ND	(16)
**10**	Yang/2014	f/74	Man.	Yes	Colon to oral/ 2 years later	Palliative	Died/ 3 months later	(17)
**11**	Baranovic/2015	m/78	Max.	Yes	Colon to oral/ 18 months later	Surgery/ palliative	Died/ 4 months later	(6)
**12**	Miyake/2015	f/65	Man.		Colon to oral/ 9 months later	Surgery/CT/ Palliative RT	Alive/ ND	(18)
**13**	Owosho/2016	m/59	Man.	ND	ND	CT	Alive/ 2 months later	(19)
**14**	Ren/2017	m/60	Man.	Yes	Colon to oral/ 2 years later	CT/RT	Alive	(4)
**15**	Ragulj/2018	f/84	Man.	No	Colon to oral/ND	CT	Died/ 20 months later	(20)
**16**	Romanet/2018	m/62	Man.	Yes	Colon to oral/ 7 years later	CT/RT	Died/ 15 months later	(21)
**17**	Di Stasio/2018	m/74	Max.	Yes	Colon to oral/ND	Surgery	Died/ND	(22)
**18**	Present case	f/69	Max.	Yes	Colon to oral/ 2 years later	CT	Died/ 6 months later	-

## Conclusion

Gingival metastasis does not necessarily have a malignant appearance. In the differential diagnosis of a rapidly growing oral lesion, a metastatic tumor is mostly proposed, even when there is no history of a primary tumor. As gingival metastasis can be the first sign of recurrence in previously treated malignancy or even the first sign for the diagnosis of a malignancy, by careful examination, a dentist can play an important role in patient survival and life expectancy by prompting the required treatment.
